# The impact of suction duration on lung collapse during one-lung ventilation

**DOI:** 10.3389/fsurg.2025.1532176

**Published:** 2025-03-31

**Authors:** Lihua Hang, Jiajun Ju, Yulin Li, Min He

**Affiliations:** Department of Anesthesiology, Kunshan Hospital Affiliated to Jiangsu University, Kunshan, China

**Keywords:** video-assisted thoracic surgery, one-lung ventilation, bronchial blockers, suction, thoracic surgery

## Abstract

**Objective:**

To investigate the effect of suction duration on lung collapse when using a bronchial blocker (BB) during single-port video-assisted thoracoscopic surgery (VATS) with one-lung ventilation (OLV).

**Methods:**

This study included 112 patients (39 males, 73 females; aged 18–75 years) with ASA physical status I or II undergoing single-port VATS under general anesthesia. Patients were randomized into four groups: control (0 s), 30 s, 60 s, and 90 s suction groups (−30 cmH₂O; *n* = 28/group). Lung collapse scores (LCS) were recorded immediately after thoracoscope entry (T0) and at 10 min (T10). The expression of nitric oxide synthase 3 (NOS-3) mRNA in lung tissue was analyzed using PCR. Lung injury pathology scores, the wet-to-dry weight ratio (W/D) of lung tissue, intraoperative hypoxemia, perioperative pulmonary complications, and use of disconnection techniques for inadequate collapse were documented.

**Results:**

At T_0_, LCS in the 30 s, 60 s, and 90 s groups were significantly higher than in the control group (*P* < 0.05), with no differences among the suction groups. At T_10_, LCS in the 60 s and 90 s groups were significantly higher than in the control group (*P* < 0.05), while no significant differences were observed between the 30 s and control groups. NOS-3 mRNA expression, lung injury pathology scores, and W/D ratios were comparable across groups. No severe hypoxemia or pulmonary complications occurred. Rescue techniques were required in four control group patients and one patient in the 30 s group but not in the 60 s and 90 s groups (*P* < 0.05).

**Conclusion:**

Suction at −30 cmH_2_O for 60 s immediately after pleural incision during one-lung ventilation with a bronchial blocker in single-port VATS significantly improves lung collapse quality without causing lung injury, making it a clinically recommended practice.

## 1 Introduction

As thoracic surgery has advanced, video-assisted thoracoscopic surgery (VATS) has become a cornerstone technique for various thoracic procedures (1). The bronchial blocker (BB) is frequently chosen for one-lung ventilation (OLV) due to its user-friendly design and lower incidence of complications like sore throat (2, 3). However, the narrower suction channel of BB can slow the deflation process of the operative lung (4). When lung collapse is insufficient, surgeons may need to resort to manual compression to enhance the surgical field, which can cause ischemia-reperfusion damage to lung tissue and elevate the risk of perioperative complications (5). Thus, optimizing the speed and effectiveness of lung collapse during OLV with BB is vital for improving patient outcomes and reducing potential lung injury. Negative pressure suction is widely used to promote lung deflation, with −30 cmH_2_O being a commonly recommended setting. Yet, the optimal duration for applying suction remains a subject of debate (6, 7). Extended suction times might increase the risk of subtle lung injuries (7). Moreover, previous research on negative pressure suction has primarily focused on three-port VATS (7). However, single-port VATS has gained increasing popularity due to its potential advantages, including reduced postoperative pain, shorter recovery times, and improved cosmetic outcomes (8). This study aims to assess the impact of suction duration under −30 cmH_2_O on lung deflation during single-port VATS with BB-assisted OLV, offering valuable guidance for refining thoracic surgical techniques.

## 2 Materials and methods

### 2.1 General data

This study was approved by the hospital's ethics committee (No.: 2022-03-014-K01), and all patients signed informed consent forms. The study included patients who underwent elective single-port VATS for lung wedge resection under general anesthesia at Kunshan First People's Hospital between September 2022 and January 2024. Inclusion criteria: patients of any gender, aged 18–75 years, with ASA physical status I or II, and no underlying cardiopulmonary diseases. Exclusion criteria: a history of thoracic surgery, forced vital capacity less than 50% of predicted value, body mass index (BMI) greater than 35 kg/m^2^, or intraoperative findings of bronchial anatomical abnormalities or pleural adhesions.

### 2.2 The sample size

The sample size was calculated based on the data from the pilot study. The mean LCS of the 0, 30 s, 60 s, and 90 s groups in the pilot study was obtained. The sample size was calculated using analysis of variance (ANOVA) in PASS11.0 (NCSS LLC, Kaysville, UT), with the standard deviation (SD) set to 1.118 (the highest SD among the four groups); *α* = 0.05, 1 - *β* = 0.8, and 25 patients were required for each group. Considering the possibility of loss to follow-up or exclusion of cases, 28 patients were calculated for each group based on a 10% withdrawal rate.

### 2.3 Group assignment and procedure

**Figure 1:**
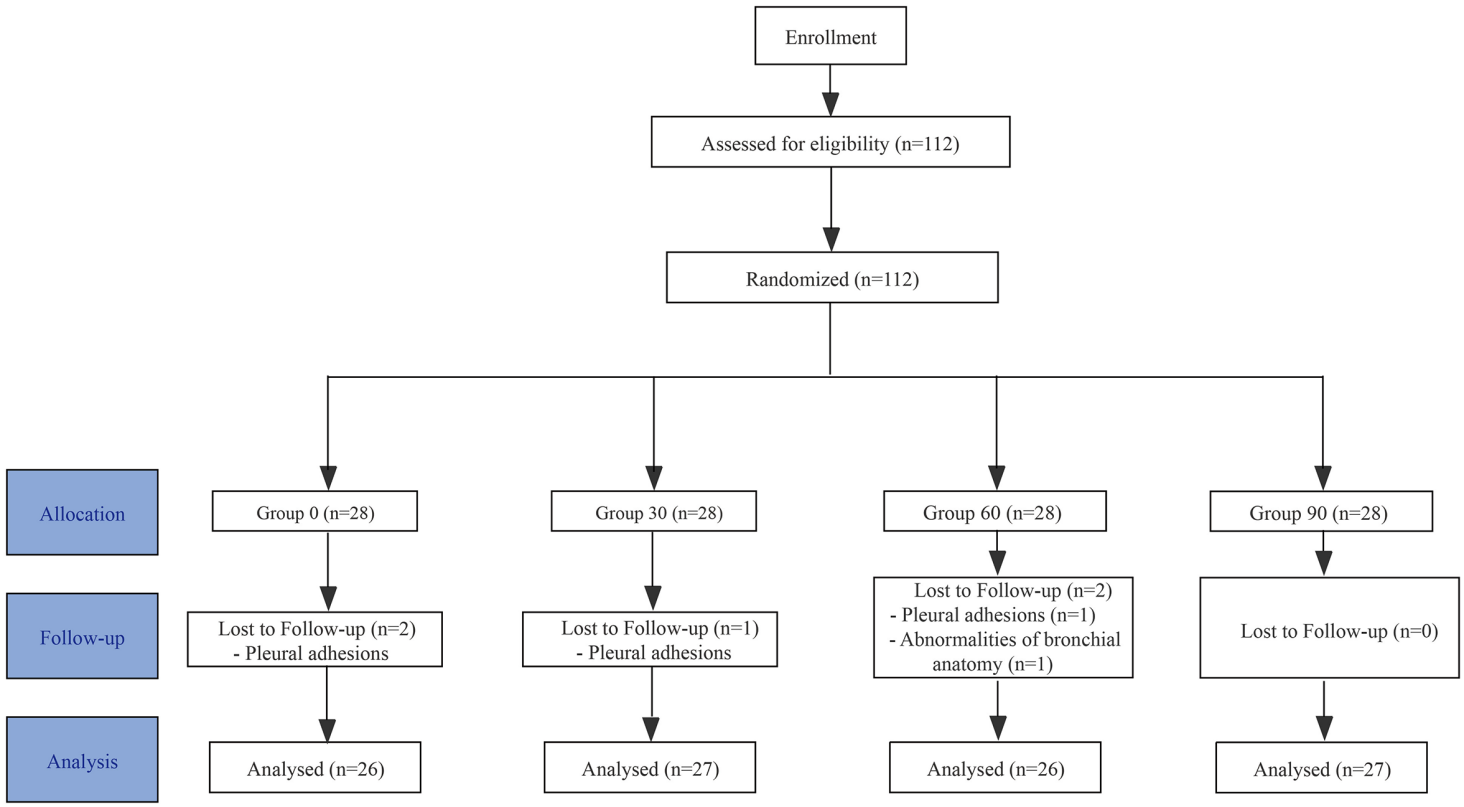
Consort statement participant flow chart.

Patients were randomly assigned to one of four groups using a random number table: control group (0 s), 30 s group, 60 s group, and 90 s group. The general surgical procedure involved opening the pleura, followed by the use of surgical instruments to compress the lung tissue and suction to optimize the surgical field for better visibility. These procedures were consistent across all experimental groups, and the same approach was used for lung manipulation, ensuring that any differences observed in lung injury pathology and the W/D ratio are likely due to the experimental conditions rather than variations in surgical technique. Each group received suction at −30 cmH_2_O for 0 s (suction device activated without actual suction), 30 s, 60 s or 90 s (Figure 1). Intraoperative personnel applying suction were blinded to the group assignments. The group allocation was concealed from the surgical team performing the procedure, ensuring that the suction duration was applied without knowledge of the specific group to avoid bias in outcome assessment.

### 2.4 Anesthesia methods

Prior to entering the operating room, all patients underwent preoperative localization in the CT room under local anesthesia and sedation. The procedure was performed using moderate anesthesia care (MAC) to ensure patient comfort. Localization was achieved using CT imaging to identify the lesion and assist in accurate positioning. After completion of preoperative localization, the patients were transferred to the operating room, where general anesthesia induction was initiated. Following this, the bronchial blocker (BB) was placed, ensuring proper alignment for the subsequent surgery.

Upon arrival in the operating room, all patients underwent routine monitoring of heart rate (HR), oxygen saturation (SpO_2_), and electrocardiography (ECG). A peripheral intravenous line was established in the forearm, and lactated Ringer's solution was administered slowly. Radial artery catheterization was performed under local anesthesia to monitor mean arterial pressure (MAP). After preoxygenation and denitrogenation (with face mask oxygenation for more than 3 min at a flow rate of 6 L/min), general anesthesia induction was initiated with intravenous administration of midazolam 0.05–0.1 mg/kg, sufentanil 0.25–0.5 μg/kg, propofol 2–2.5 mg/kg, and succinylcholine 1.5 mg/kg. Tracheal intubation was performed using a single-lumen endotracheal tube (7.5 mm for males, 7.0 mm for females) under video laryngoscopy, followed by mechanical ventilation using a volume-controlled ventilation mode. Prior to the initiation of OLV, the respiratory settings were fraction of inspired oxygen (FiO_2)_100%, tidal volume 6–8 ml/kg, with respiratory rate adjusted to maintain end-tidal CO_2_ (PETCO_2_) between 35 and 45 mmHg.

Anesthesia maintenance involved continuous intravenous infusion of propofol 100–180 μg/kg/min, remifentanil 0.05–0.4 μg/kg/min, and dexmedetomidine 0.2–0.7 μg/kg/min, with intermittent boluses of cisatracurium 0.05 mg/kg to maintain muscle relaxation. After placing the patient in the lateral decubitus position, the bronchial blocker (BB; Hangzhou Tappa Medical Technology Co., Hangzhou, China) was inserted into the endotracheal tube under fiberoptic bronchoscopy guidance, with the Tappa angle facing upward, to initiate OLV. The BB's suction channel remained closed until pleural incision. During OLV, FiO_2_ was set to 50% and tidal volume to 4–6 ml/kg. After the surgeon inserted the chest drainage tube, the BB was removed, and bilateral lungs were suctioned and reinflated. Two-lung ventilation was resumed, and 50 mg flurbiprofen was administered intravenously. Dexmedetomidine infusion was discontinued 30 min before the end of surgery, and propofol and remifentanil were stopped at the end of the procedure. Intraoperative adjustments to anesthetic drug dosages were made based on hemodynamic parameters, ensuring MAP and HR fluctuations remained within 30% of baseline values, with vasoactive agents administered as necessary. Postoperatively, patients were awakened in the post-anesthesia care unit (PACU), and extubation of the single-lumen endotracheal tube was performed once extubation criteria were met.

### 2.5 Quantitative real-time PCR (qRT-PCR)

Total RNA from lung tissue was extracted using the Trizol method according to the manufacturer's instructions. The concentration and purity of the RNA were determined using a NanoDrop spectrophotometer. cDNA was synthesized from total RNA using a reverse transcription kit, and PCR amplification was performed using cDNA as a template. Glyceraldehyde-3-phosphate dehydrogenase (GAPDH, HUMAN) was used as the reference gene, and the relative expression of the target gene NOS-3 mRNA was calculated using the 2^−ΔΔCt^ method (9). To ensure objectivity, both the RNA extraction and subsequent analysis were performed by independent researchers who were blinded to the experimental groups.

### 2.6 Hematoxylin and eosin (HE) staining

After rewarming, lung tissues were fixed in 4% paraformaldehyde, followed by paraffin embedding and sectioning (5 μm thickness) for HE staining. Pathological changes in the lung tissues were observed under a light microscope by a pathologist. Lung injury scores were assessed according to the method described by Xu et al. (10). The scoring criteria used to assess lung injury included alveolar fibrin/edema, alveolar hemorrhage, septal thickening, and cellular infiltration. Each parameter was graded on a scale from 0 to 3. A score of 0 indicated no injury, while scores of 1, 2, and 3 represented the involvement of less than 25%, 25%–50%, and more than 50% of the alveolar region, respectively.

### 2.7 Lung wet-to-dry weight ratio (W/D)

After rewarming, the lung tissues were blotted to remove surface moisture and immediately weighed to obtain the wet weight (W). The tissues were then dried in an oven at 70°C until a constant weight was achieved, and the dry weight (D) was recorded. The W/D ratio was calculated by dividing the wet weight by the dry weight (11).

### 2.8 Observation indicators

Lung collapse scores (LCS) were recorded at 0 min (T_0_) and 10 min (T_10_) after thoracoscope entry into the chest cavity. Two independent surgeons, blinded to group assignments, assessed LCS via video recordings using the visual analog scale (VAS). The VAS is one of the most used methods for evaluating the quality of lung collapse, where 0 represents no lung collapse and 10 represents complete lung collapse (7, 12). The lung collapse scoring was performed independently by both surgeons, and their results were compared to ensure consistency. Both assessors were blinded to the experimental groups during evaluation.

Implementation of Rescue Measures: If, upon thoracoscope entry, the surgeons observed poor lung collapse quality and inadequate operating space that hindered the procedure, a disconnection technique was employed to improve lung collapse. The steps were as follows: (1) Adjust the ventilator to manual mode and fully open the pressure relief valve. (2) Deflate the BB cuff. (3) After 1 min, reinflate the cuff and resume OLV (3).

Additionally, the following variables were recorded: NOS-3 mRNA expression levels, pathological lung injury scores, and the wet-to-dry weight ratio (W/D) of lung tissue. Perioperative pulmonary complications (such as pulmonary edema and respiratory failure) were noted, as well as any occurrence of intraoperative severe hypoxemia (defined as SpO_2_ <90% requiring CPAP or bilateral lung ventilation). The following surgery-related times were also documented: pre-OLV time (defined as the time from OLV initiation to pleural incision), OLV time (defined as the time from OLV initiation to bilateral lung ventilation after lung reinflation), skin incision time (defined as the time from skin incision to pleural incision), trocar insertion time (defined as the time from pleural incision to thoracoscope entry), and total surgery time (defined as the time from skin incision to the completion of suturing).

### 2.9 Statistical analysis

Based on the results of the preliminary experiment, the average LCS at T0 for the s, 30 s, 60 s, and 90 s groups were 3.5, 3.7, 4.5, and 5, respectively (with three patients per group), and the largest standard deviation among the four groups was 1.7. Assuming a significance level of *α* = 0.05 and a power of 1 - *β* = 0.8, and considering a 20% dropout rate, the required sample size was calculated to be 112 patients, with 28 patients in each group, using PASS 11.0 software (13).

Data analysis was performed using SPSS 26.0 software. Continuous variables with a normal distribution were expressed as mean ± standard deviation (x̅ ± s), and intergroup comparisons were conducted using one-way analysis of variance (ANOVA). For non-normally distributed variables, data were expressed as median and interquartile range [M (IQR)], and comparisons between groups were made using non-parametric tests. Categorical data were presented as frequencies (*n*, %), and comparisons between groups were performed using the chi-square (χ^2^) test or Fisher's exact test. A *P*-value of less than 0.05 was considered statistically significant.

## 3 Results

A total of 112 patients were initially enrolled in this study. After excluding 4 patients with pleural adhesions and 1 patient with tracheobronchial variation (right upper lobe bronchus anomaly), 107 patients were included for analysis. The patients were divided into four groups: 26 in the 0 s group, 27 in the 30 s group, 26 in the 60 s group, and 28 in the 90 s group. There were no statistically significant differences in age, sex, BMI, or ASA classification among the four groups (Table 1).

**Table 1:** Comparison of general characteristics Among the four groups.

Group	*N*	Age (years)	Male/Female (cases)	BMI (kg/m^2^)	ASA I/II (cases)
0 s	26	47.2 ± 11.4	7/19	22.3 ± 2.9	11/15
30 s	27	43.8 ± 9.0	10/17	24.2 ± 3.2	15/12
60 s	26	48.3 ± 12.5	10/16	23.0 ± 3.3	12/14
90 s	28	45.4 ± 13.3	9/19	23.0 ± 3.7	9/19

Data are expressed in mean (standard deviation) or number (%).

BMI, body mass index; ASA, American Society of Anesthesiologists.

Additionally, no significant differences were observed in the surgical side, preoperative lung nodule localization, pre-OLV time, OLV duration, skin incision time, trocar insertion time, or total operative time across the groups (Table 2).

**Table 2:** Comparison of surgery-related parameters Among the four groups.

Parameter	0 s group (*n* = 26)	30 s group (*n* = 27)	60 s group (*n* = 26)	90 s group (*n* = 28)
Left/right surgical side (cases)	12/14	13/14	15/11	9/19
Preoperative lung nodule localization (cases)	8	9	10	9
Pre-OLV time (min)	6.7 ± 1.9	7.3 ± 2.0	7.2 ± 2.7	8.1 ± 3.0
OLV duration (min)	44.8 ± 10.7	39.1 ± 13.1	43.2 ± 18.0	43.9 ± 19.7
Skin incision time (min)	1.6 ± 0.8	1.8 ± 0.9	1.7 ± 0.6	2.0 ± 1.0
Trocar insertion time (min)	1.2 ± 0.4	1.1 ± 0.5	1.1 ± 0.5	1.0 ± 0.3
Total surgery time (min)	44.8 ± 10.6	38.6 ± 12.8	42.7 ± 17.5	42.8 ± 19.0

**Figure 2:**
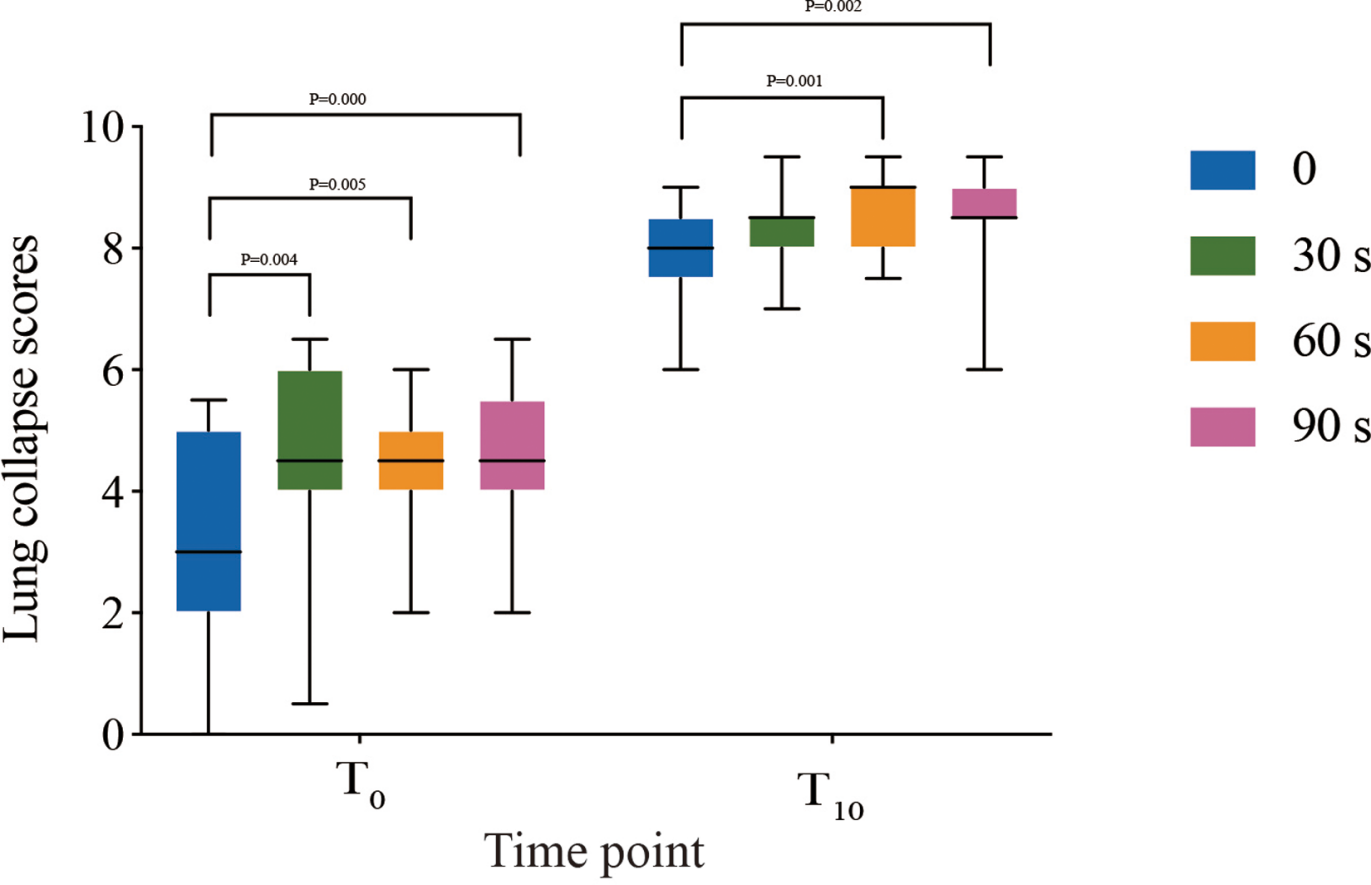
Lung collapse scores of 0 min and 10 min on a 10-point visual analogue scale after the visualization of the lung. The data are presented as medians, ranges, and interquartile ranges (25th–75th percentile). Comparison of lung collapse scores of 0 min and 10 min in 4 groups analyzed by using with Kruskal–Wallis test, the results of the pairwise comparison analyzed by the Mann–Whitney *U*-test. * Point: *P* < 0.05.

At time point T_0_, there were significant differences in LCS between the groups (*P* < 0.05); the LCS values were significantly higher in the 30 s, 60 s, and 90 s groups compared to the 0 s group (*P* < 0.05), with no significant differences between the 30 s, 60 s, and 90 s groups (*P* > 0.05). At time point T_10_, significant differences in LCS were still observed among the groups (*P* < 0.05); the 60 s and 90 s groups had significantly higher LCS than the 0 s group (*P* < 0.05), while no significant difference was found between the 0 s and 30 s groups (*P* > 0.05), or between the 60 s and 90 s groups (*P* > 0.05) (Table 3; Figure 2). In the 0 s and 30 s groups, 4 and 1 patients, respectively, required the use of a disconnection technique to accelerate lung collapse due to poor lung deflation, whereas no rescue measures were needed in the 60 s and 90 s groups; this difference was statistically significant when compared to the 0 s group (*P* < 0.05) (Table 3).

**Table 3:** Comparison of LCS among the four groups.

Group	*N*	T_0_ LCS (points)	T_10_ LCS (points)	Disconnection required (cases)
0 s	26	3.0 (2.0–5.0)	8.0 (7.5–8.5)	4
30 s	27	4.5 (4.0–5.0)*	8.5 (8.0–8.5)	1
60 s	26	4.5 (4.0–5.0)*	9.0 (8.0–9.0)*	0*
90 s	28	4.5 (4.0–5.5)*	8.5 (8.5–9.0)*	0*

Compared with the 0-s group.

* *P* < 0. 05.

No cases of severe hypoxemia or perioperative pulmonary complications were observed in any of the groups. Finally, there were no significant differences in NOS-3 mRNA expression, lung injury pathology scores, or lung wet-to-dry weight ratio between the 0 s and 90 s groups (Table 4).

**Table 4:** Comparison of NOS-3 mRNA expression, lung injury pathology scores, and lung W/D ratio between the 0 s and 90 s groups.

Group	*N*	NOS-3 mRNA expression	Lung injury pathology score (points)	Lung W/D ratio
0 s	4	1.4 ± 0.9	0.5 ± 0.2	4.1 ± 0.7
90 s	4	1.2 ± 0.9	0.6 ± 0.2	4.3 ± 0.3

## 4 Discussion

BB is a commonly used lung isolation device in VATS. However, due to the narrow and long structure of the BB exhaust tube, the residual gas in the non-ventilated lung is expelled relatively slowly. Achieving high-quality lung collapse on the operative side is critical for the success of thoracic surgery. When using BB for OLV, improving the quality of lung collapse is essential for enhancing surgical safety and reducing postoperative lung injury.

Negative pressure suction is one of the common methods used to accelerate lung collapse, though the reported suction times vary across studies. Quan et al. (7) demonstrated that, when using a double-lumen tube (DLT) for OLV, applying suction at −30 cmH₂O for 60 s immediately after pleural incision significantly improved LCS at various intraoperative time points. Similarly, El-Tahan et al. (6) found that using the Arndt BB for OLV, applying suction for 90 s after draping significantly improved lung collapse quality.

In this study, both the 60 s and 90 s groups showed significantly improved lung collapse quality compared to the control (0 s group) and 30 s group, with no evidence of occult lung injury. However, we observed no statistically significant difference in LCS between the 60 s and 90 s groups, suggesting that the increased suction time beyond 60 s may not provide additional benefits in improving lung collapse. These results are consistent with findings from studies using DLT for OLV, where longer suction durations did not consistently lead to further improvements in lung collapse quality (7).

Moreover, the choice of OLV device may influence the effectiveness of suction in achieving adequate lung collapse. DLT provides more precise separation of the lungs, but its use is associated with a higher risk of airway trauma and a more invasive procedure (8). In contrast, Arndt BB, while less invasive, has been shown to be equally effective in achieving lung collapse, especially in single-port VATS. The BB is associated with fewer complications and less risk of airway injury (3), making it a favorable option in many cases, especially for less invasive thoracic surgeries.

Our study suggests that 60 s of suction at −30 cmH₂O following pleural incision is the optimal duration for accelerating lung collapse when using the Arndt BB for single-port VATS. This is consistent with previous research showing that shorter suction durations (e.g., 30–60 s) can achieve similar outcomes in terms of lung collapse quality, but prolonged suction times may increase the risk of lung injury without providing additional benefits. By identifying the optimal suction time, our study provides important insights into balancing effective lung collapse with minimizing complications during thoracic procedures.

Most previous studies on negative pressure suction were based on three-port VATS, while this study involved patients undergoing single-port VATS. With the development of thoracic surgery techniques, single-port VATS has gradually replaced three-port VATS due to its advantages of less trauma, reduced postoperative pain, and faster recovery (8). The time required from skin incision to the completion of the three-port setup in three-port VATS is approximately 10 min, whereas single-port VATS requires only about 90 s (6), Compared to three-port VATS, single-port VATS offers shorter procedural times, and surgeons can begin thoracic operations immediately after placing a single trocar. Therefore, greater attention is given to the quality of lung collapse immediately after thoracoscope entry.

Prolonged suction duration may result in lung injury. Studies have shown that continuous suction at a pressure of −272 cmH₂O for 160 s can cause ulceration and necrosis of tracheal tissue (14). Although previous studies on negative pressure suction have not observed severe intraoperative hypoxemia or perioperative pulmonary complications, the potential for negative pressure suction to induce occult lung injury remains a primary concern for anesthesiologists. Current research suggests that suction durations aimed at accelerating lung collapse are typically less than 90 s. Therefore, the maximum suction time in this study was set to 90 s. To assess the presence of occult lung injury, we measured NOS-3 mRNA expression, pathological lung injury scores, and the lung (W/D) ratio. NOS-3 is a subtype of nitric oxide synthase in humans, and its increased expression significantly promotes the production of nitric oxide (NO) (15). Elevated NO levels play an important role in reducing pulmonary vascular tension and alleviating lung injury (16). Hence, NOS-3 is considered a key biomarker for evaluating lung injury. Additionally, the lung W/D ratio is the gold standard for assessing pulmonary edema (11). while histopathological sections of lung tissue can reveal the presence of red blood cell aggregation and edema fluid in the alveolar cavity (10), serving as critical indicators of lung injury and pulmonary edema. The results of this study showed no statistically significant differences in NOS-3 mRNA expression, pathological lung injury scores, or lung W/D ratio between the 0 s group and the 90 s group. Therefore, even with a suction time of 90 s, negative pressure suction at −30 cmH₂O is still a safe method for accelerating lung collapse.

Techniques such as bronchial blockade, early initiation of OLV, oxygen insufflation, and disconnection methods are commonly used in clinical practice to accelerate lung collapse (15, 17, 18). Somma et al. (19) proposed an optimized approach for enhancing lung collapse using a DLT during OLV, incorporating early OLV, bronchial blockade, and disconnection techniques. Building upon this, our study further optimized the process by combining oxygen insufflation, early OLV, bronchial blockade, and negative pressure suction to provide surgeons with an improved surgical field. During the period from patient arrival to the initiation of OLV, the FiO_2_ was set to 100% to replace the nitrogen (N_2_) with a lower blood solubility in the operative lung (20). After the patient was placed in the lateral decubitus position, OLV was initiated early, and the exhaust port of the BB was occluded to prevent “gas exchange” (i.e., passive ventilation) between the operative lung and the surrounding air (21). Upon pleural incision, residual gas in the operative lung was rapidly evacuated using negative pressure. The disconnection technique, also known as the apnea technique, was employed as a remedial measure in cases where the surgeon observed poor lung collapse after thoracoscopic entry into the thoracic cavity.

In this study, one patient with a tracheobronchial anomaly (abnormal opening of the right upper lobe bronchus) was excluded. Reports of patients with bronchial anatomical variations undergoing OLV have been documented both domestically and internationally (22). In such patients, the opening of the right upper lobe bronchus is located above the carina, and inflation of the BB cuff fails to completely block the non-ventilated lung, leading to unsuccessful OLV. For these patients, selecting a left DLT may be more suitable.

This study has certain limitations, including the use of the VAS to assess the quality of lung collapse. Although this method is highly correlated with clinical practice, it lacks objectivity. Currently, no objective and quantitative criteria exist for evaluating lung collapse quality, which warrants further research. Future studies could explore the use of advanced imaging techniques to provide more objective, reproducible, and quantitative measurements of lung collapse. For instance, computed tomography (CT) or magnetic resonance imaging (MRI) could be employed to assess the degree of lung collapse with high resolution and accuracy. Additionally, optical imaging or fluorescence-based techniques may allow for real-time, *in vivo* visualization of lung collapse dynamics, offering a more accurate assessment.

Excluding patients with anatomical bronchial abnormalities was necessary to ensure a homogeneous study population and minimize confounding factors. However, this limits the generalizability of our findings. Anatomical variations, such as bronchial malformations, can influence the effectiveness of OLV techniques, including those used for lung collapse. Patients with such abnormalities may experience different outcomes, which were not assessed in this study. Future research should investigate the impact of these variations on lung collapse, possibly through stratified analyses or by including a more diverse patient population. Advanced imaging techniques to evaluate bronchial anatomy could provide deeper insights into how these conditions affect lung collapse and OLV management.

In Conclusion, the use of BB for OLV in single-port VATS, combined with suction at −30 cmH₂O for 60 s immediately after pleural incision, can significantly improve the quality of lung collapse without increasing the risk of potential lung injury, making it a valuable approach for clinical application.

## Data Availability

The original contributions presented in the study are included in the article/Supplementary Material, further inquiries can be directed to the corresponding author.
